# Supplementary material for: Modeling the diverse effects of divisive normalization on noise correlations
Source: PLoS Comput Biol. 2023 Nov 30;19(11):e1011667. doi: 10.1371/journal.pcbi.1011667 (PMC10715670; doi:10.1371/journal.pcbi.1011667)
Supplement: S1 Text — Moments of the Ratio of Gaussians distribution for the general case of cross-correlations between numerator and denominator. (PDF) [file pcbi.1011667.s001.pdf]

S1 Text

Derivation of Moments for the Generalized Model

(see the subsection Generative Model – Pairwise Ratio of Gaussians (RoG) in the Methods section of the main text)

Throughout this paper, we assumed that the numerator and denominator variables are uncorrelated, allowing us to consider two separate variables $\boldsymbol{N}_t, \boldsymbol{D}_t$ to be bivariate Gaussians. In the generalized form of the pairwise RoG generative model, we consider $(\boldsymbol{N}_t, \boldsymbol{D}_t)$ to be distributed according to a four-dimensional Gaussian:

$$\begin{pmatrix} \boldsymbol{N}_t \\ \boldsymbol{D}_t \end{pmatrix} = \begin{pmatrix} N_{1,t} \\ N_{2,t} \\ D_{1,t} \\ D_{2,t} \end{pmatrix} \sim \mathcal{N}\left(\boldsymbol{\mu}_{\boldsymbol{N},\boldsymbol{D}} = \begin{pmatrix} \mu_{N_1} \\ \mu_{N_2} \\ \mu_{D_1} \\ \mu_{D_2} \end{pmatrix}, \boldsymbol{\Sigma}_{\boldsymbol{N},\boldsymbol{D}} \right)$$

$$\boldsymbol{\Sigma}_{\boldsymbol{N},\boldsymbol{D}} = \begin{pmatrix} \sigma_{N_1}^2 & \rho_N \sigma_{N_1}\sigma_{N_2} & 0 & \rho_{N_1,D_2}\sigma_{N_1}\sigma_{D_2} \\ \rho_N \sigma_{N_1}\sigma_{N_2} & \sigma_{N_2}^2 & \rho_{N_2,D_1}\sigma_{N_2}\sigma_{D_1} & 0 \\ 0 & \rho_{N_2,D_1}\sigma_{N_2}\sigma_{D_1} & \sigma_{D_1}^2 & \rho_D \sigma_{D_1}\sigma_{D_2} \\ \rho_{N_1,D_2}\sigma_{N_1}\sigma_{D_2} & 0 & \rho_D \sigma_{D_1}\sigma_{D_2} & \sigma_{D_2}^2 \end{pmatrix} \tag{S1.1}$$

$$\boldsymbol{\eta}_t \sim \mathcal{N}(\boldsymbol{\mu}_{\boldsymbol{\eta}}, \boldsymbol{\Sigma}_{\boldsymbol{\eta}})$$

$$\boldsymbol{R}_t = (R_1, R_2)_t = f_{\div}(\boldsymbol{N}_t, \boldsymbol{D}_t) + \boldsymbol{\eta}_t = \frac{\boldsymbol{N}_t}{\boldsymbol{D}_t} + \boldsymbol{\eta}_t$$

We have assumed that correlations within the equivalent independent RoG model (i.e., the correlations between N_1, D_1 and between N_2, D_2), based on our prior work that showed overfitting when including these correlations [1].

Then, we consider the Taylor expansion around $\boldsymbol{\mu}_{\boldsymbol{N},\boldsymbol{D}}$, as in the main text and discarding higher order terms. The mean of the ratio distribution is the same as previously derived as

Eq (4). However, the derivation for the covariance is slightly different:

$$\begin{aligned}\boldsymbol{\Sigma}_{\boldsymbol{R}} &= \mathrm{E}[(\boldsymbol{R}-\boldsymbol{\mu}_{\boldsymbol{R}})(\boldsymbol{R}-\boldsymbol{\mu}_{\boldsymbol{R}})^\top] \\ &\approx \nabla f_{\div}|_{\boldsymbol{\mu}_{\boldsymbol{N}},\boldsymbol{\mu}_{\boldsymbol{D}}} \mathrm{E}\left[\left(\begin{pmatrix}\boldsymbol{N}\\ \boldsymbol{D}\end{pmatrix} - \begin{pmatrix}\boldsymbol{\mu}_{\boldsymbol{N}}\\ \boldsymbol{\mu}_{\boldsymbol{D}}\end{pmatrix}\right)\left(\begin{pmatrix}\boldsymbol{N}\\ \boldsymbol{D}\end{pmatrix} - \begin{pmatrix}\boldsymbol{\mu}_{\boldsymbol{N}}\\ \boldsymbol{\mu}_{\boldsymbol{D}}\end{pmatrix}\right)^\top\right] \nabla f_{\div}|^\top_{\boldsymbol{\mu}_{\boldsymbol{N}},\boldsymbol{\mu}_{\boldsymbol{D}}} + \boldsymbol{\Sigma}_{\boldsymbol{\eta}} \\ &\approx \nabla f_{\div}|_{\boldsymbol{\mu}_{\boldsymbol{N}},\boldsymbol{\mu}_{\boldsymbol{D}}} \boldsymbol{\Sigma}_{\boldsymbol{N},\boldsymbol{D}} \nabla f_{\div}|^\top_{\boldsymbol{\mu}_{\boldsymbol{N}},\boldsymbol{\mu}_{\boldsymbol{D}}} + \boldsymbol{\Sigma}_{\boldsymbol{\eta}} \end{aligned} \tag{S1.2}$$

Note that, instead of the block diagonal matrix used in Eq (5), we use the full covariance matrix defined in Eq (S1.1). The formula for the variance is the same as previously derived. For the covariance and correlation, we have:

$$\mathrm{Cov}\,(R_1, R_2) \approx \frac{\mu_{N_1}\mu_{N_2}}{\mu_{D_1}\mu_{D_2}}\left(\rho_N \delta_{N_1}\delta_{N_2} + \rho_D \delta_{D_1}\delta_{D_2} + \rho_{N_1,D_2}\delta_{N_1}\delta_{D_2} + \rho_{N_2,D_1}\delta_{N_2}\delta_{D_1} + \rho_\eta \widehat{\sigma_{\eta_1}}\widehat{\sigma_{\eta_2}}\right) \tag{S1.3}$$

$$\mathrm{Corr}(R_1, R_2) \approx \frac{\rho_N \delta_{N_1}\delta_{N_2} + \rho_D \delta_{D_1}\delta_{D_2} + \rho_{N_1,D_2}\delta_{N_1}\delta_{D_2} + \rho_{N_2,D_1}\delta_{N_2}\delta_{D_1} + \rho_\eta \widehat{\sigma_{\eta_1}}\widehat{\sigma_{\eta_2}}}{\sqrt{\delta^2_{N_1} + \delta^2_{D_1} + \widehat{\sigma_{\eta_1}}^2}\sqrt{\delta^2_{N_2} + \delta^2_{D_2} + \widehat{\sigma_{\eta_2}}^2}} \tag{S1.4}$$

This adds two additional parameters ($\rho_{N_1,D_2}, \rho_{N_2,D_1}$) that need to be optimized using the negative log-likelihood (Eq (11)). We note that the accompanying code toolbox includes these parameters.

References

[1] Coen-Cagli R, Solomon SS. Relating Divisive Normalization to Neuronal Response Variability. The Journal of Neuroscience. 2019;39(37):7344–7356. doi:10.1523/JNEUROSCI.0126-19.2019.
