# Supplementary material for: Modeling the diverse effects of divisive normalization on noise correlations
Source: PLoS Comput Biol. 2023 Nov 30;19(11):e1011667. doi: 10.1371/journal.pcbi.1011667 (PMC10715670; doi:10.1371/journal.pcbi.1011667)
Supplement: S2 Text — Details of the calculation of the negative-log likelihood for the Ratio of Gaussians distribution. (PDF) [file pcbi.1011667.s002.pdf]

S2 Text

Derivation of Negative Log-Likelihood for the Model

(see the subsection Fitting the RoG to Data in the Methods section of the main text)

The model assumptions that the data is distributed according to a bivariate Gaussian distribution with mean and covariance given by the RoG approximation (Eqs (4) and (5) in the main text). From this, we calculate the negative log-likelihood:

$$
\begin{aligned}
-\log p(\{\boldsymbol{R}_t(s)\};\Theta) &= -T\sum_{s\in S}\left\langle \log\mathcal{N}(\boldsymbol{R}_t(s);\boldsymbol{\mu}_{\boldsymbol{R}}(s,\Theta),\boldsymbol{\Sigma}_{\boldsymbol{R}}(s,\Theta))\right\rangle_T \qquad \text{(S2.1)}\\
&\approx \frac{T}{2}\sum_{s\in S}\Big\{\log|\boldsymbol{\Sigma}_{\boldsymbol{R}}(s,\Theta)| + \left\langle(\boldsymbol{R}_t(s)-\boldsymbol{\mu}_{\boldsymbol{R}}(s,\Theta))^\top\boldsymbol{\Sigma}_{\boldsymbol{R}}^{-1}(s,\Theta)(\boldsymbol{R}_t(s)-\boldsymbol{\mu}_{\boldsymbol{R}}(s,\Theta))\right\rangle_T\Big\}\\
&\approx \frac{T}{2}\sum_{s\in S}\Big\{\log|\boldsymbol{\Sigma}_{\boldsymbol{R}}(s,\Theta)| + \overbrace{\left\langle\boldsymbol{R}_t(s)^\top\boldsymbol{\Sigma}_{\boldsymbol{R}}^{-1}(s,\Theta)\boldsymbol{R}_t(s)\right\rangle_T}^{*} - \left\langle\boldsymbol{R}_t(s)^\top\right\rangle_T\boldsymbol{\Sigma}_{\boldsymbol{R}}^{-1}(s,\Theta)\boldsymbol{\mu}_{\boldsymbol{R}}(s,\Theta)\\
&\qquad - \boldsymbol{\mu}_{\boldsymbol{R}}(s,\Theta)^\top\boldsymbol{\Sigma}_{\boldsymbol{R}}^{-1}(s,\Theta)\left\langle\boldsymbol{R}_t(s)\right\rangle_T + \boldsymbol{\mu}_{\boldsymbol{R}}(s,\Theta)^\top\boldsymbol{\Sigma}_{\boldsymbol{R}}^{-1}(s,\Theta)\boldsymbol{\mu}_{\boldsymbol{R}}(s,\Theta)\Big\}\\
&\approx \frac{T}{2}\sum_{s\in S}\Big\{\log|\boldsymbol{\Sigma}_{\boldsymbol{R}}(s,\Theta)| + \overbrace{\mathrm{Tr}(\boldsymbol{\Sigma}_{\boldsymbol{R}}^{-1}(s,\Theta)\widehat{\boldsymbol{\Sigma}_{\boldsymbol{R}}}(s)) + \widehat{\boldsymbol{\mu}_{\boldsymbol{R}}}(s)^\top\boldsymbol{\Sigma}_{\boldsymbol{R}}^{-1}(s,\Theta)\widehat{\boldsymbol{\mu}_{\boldsymbol{R}}}(s)}^{*} \qquad \text{(S2.2)}\\
&\qquad - \boldsymbol{\mu}_{\boldsymbol{R}}(s,\Theta)^\top\boldsymbol{\Sigma}_{\boldsymbol{R}}^{-1}(s,\Theta)\widehat{\boldsymbol{\mu}_{\boldsymbol{R}}}(s) - \widehat{\boldsymbol{\mu}_{\boldsymbol{R}}}(s)^\top\boldsymbol{\Sigma}_{\boldsymbol{R}}^{-1}(s,\Theta)\boldsymbol{\mu}_{\boldsymbol{R}}(s,\Theta)\\
&\qquad + \boldsymbol{\mu}_{\boldsymbol{R}}(s,\Theta)^\top\boldsymbol{\Sigma}_{\boldsymbol{R}}^{-1}(s,\Theta)\boldsymbol{\mu}_{\boldsymbol{R}}(s,\Theta)\Big\}
\end{aligned}
$$

The terms marked by $*$ are equal according to:

$$
\begin{aligned}
\left\langle\boldsymbol{R}_t(s)^\top\boldsymbol{\Sigma}_{\boldsymbol{R}}^{-1}(s,\Theta)\boldsymbol{R}_t(s)\right\rangle_T &= \mathrm{Tr}\Big(\left\langle\boldsymbol{R}_t(s)^\top\boldsymbol{\Sigma}_{\boldsymbol{R}}^{-1}(s,\Theta)\boldsymbol{R}_t(s)\right\rangle_T\Big) = \mathrm{Tr}\Big(\boldsymbol{\Sigma}_{\boldsymbol{R}}^{-1}(s,\Theta)\left\langle\boldsymbol{R}_t(s)^\top\boldsymbol{R}_t(s)\right\rangle_T\Big)\\
&= \mathrm{Tr}\Big(\boldsymbol{\Sigma}_{\boldsymbol{R}}^{-1}(s,\Theta)(\widehat{\boldsymbol{\Sigma}_{\boldsymbol{R}}}(s) + \widehat{\boldsymbol{\mu}_{\boldsymbol{R}}}(s)\widehat{\boldsymbol{\mu}_{\boldsymbol{R}}}(s)^\top)\Big)\\
&= \mathrm{Tr}(\boldsymbol{\Sigma}_{\boldsymbol{R}}^{-1}(s,\Theta)\widehat{\boldsymbol{\Sigma}_{\boldsymbol{R}}}(s)) + \mathrm{Tr}\Big(\boldsymbol{\Sigma}_{\boldsymbol{R}}^{-1}(s,\Theta)\widehat{\boldsymbol{\mu}_{\boldsymbol{R}}}(s)\widehat{\boldsymbol{\mu}_{\boldsymbol{R}}}(s)^\top)\Big)\\
&= \mathrm{Tr}(\boldsymbol{\Sigma}_{\boldsymbol{R}}^{-1}(s,\Theta)\widehat{\boldsymbol{\Sigma}_{\boldsymbol{R}}}(s)) + \widehat{\boldsymbol{\mu}_{\boldsymbol{R}}}(s)^\top\boldsymbol{\Sigma}_{\boldsymbol{R}}^{-1}(s,\Theta)\widehat{\boldsymbol{\mu}_{\boldsymbol{R}}}(s)
\end{aligned}
$$

Using the fact that $\mathrm{Tr}(AB) = \mathrm{Tr}(BA)$ and $c = \mathrm{Tr}(c)$ for A, B matrices and c a scalar. Simplifying Eq (S2.2) produces Eq (11) in the main text.
