# Supplementary material for: Modeling the diverse effects of divisive normalization on noise correlations
Source: PLoS Comput Biol. 2023 Nov 30;19(11):e1011667. doi: 10.1371/journal.pcbi.1011667 (PMC10715670; doi:10.1371/journal.pcbi.1011667)
Supplement: S3 Text — Expands on Inference of single trial normalization from measured neural activity, Eq (14), showing the coefficient expressions. (PDF) [file pcbi.1011667.s003.pdf]

S3 Text

Negative Log-Posterior for Inference of Single-Trial Normalization Strength

(see the subsection Inference of Single Trial Normalization from Measured Neural Activity in the Methods section of the main text)

Using Bayes' theorem and the change of variables formula, we compute the negative log of the probability distribution of the single-trial normalization signals ($\boldsymbol{D}_t$) given the observed neural activity during that trial ($\boldsymbol{R}_t$) as:

$$-\log p(\boldsymbol{D}_t|\boldsymbol{R}_t) = -\log|D_1 D_2| - \log p(N_1, N_2) - \log p(D_1, D_2) \tag{S3.1}$$

We use the generative model (Eq (2)) and simplify to obtain the following expression:

$$\begin{aligned}
-\log p(\boldsymbol{D}_t|\boldsymbol{R}_t) = &-\log|D_1 D_2| \\
&+ \frac{1}{2}\Bigg[D_1^2 \left(\frac{R_1^2}{\sigma_{N_1}^2 (1-\rho_N^2)} + \frac{1}{\sigma_{D_1}^2 (1-\rho_D^2)}\right) && \text{(A1)} \\
&+ D_1 \left(\frac{-2\mu_{N_1} R_1}{\sigma_{N_1}^2 (1-\rho_N^2)} + \frac{2\rho_N \mu_{N_2} R_1}{\sigma_{N_1}\sigma_{N_2} (1-\rho_N^2)} - \frac{2\mu_{D_1}}{\sigma_{D_1}^2 (1-\rho_D^2)} + \frac{2\rho_D \mu_{D_2}}{\sigma_{D_1}\sigma_{D_2} (1-\rho_D^2)}\right) && \text{(B1)} \\
&+ D_2^2 \left(\frac{R_2^2}{\sigma_{N_2}^2 (1-\rho_N^2)} + \frac{1}{\sigma_{D_2}^2 (1-\rho_D^2)}\right) && \text{(A2)} \\
&+ D_2 \left(\frac{-2\mu_{N_2} R_2}{\sigma_{N_2}^2 (1-\rho_N^2)} + \frac{2\rho_N \mu_{N_1} R_2}{\sigma_{N_1}\sigma_{N_2} (1-\rho_N^2)} - \frac{2\mu_{D_2}}{\sigma_{D_2}^2 (1-\rho_D^2)} + \frac{2\rho_D \mu_{D_1}}{\sigma_{D_1}\sigma_{D_2} (1-\rho_D^2)}\right) && \text{(B2)} \\
&+ D_1 D_2 \left(-\frac{2\rho_N R_1 R_2}{\sigma_N \sigma_{N_2} (1-\rho_N^2)} - \frac{2\rho_D}{\sigma_{D_1}\sigma_{D_2} (1-\rho_D^2)}\right)\Bigg] && \text{(C)}
\end{aligned}$$

Substituting the coefficients on the right, we can write this equation as:

$$-\log p\left(\boldsymbol{D}_t|\boldsymbol{R}_t\right) \propto -\log\left|D_1 D_2\right| + \frac{1}{2}\left(A_1 {D_1}^2 + B_1 D_1 + A_2 {D_2}^2 + B_2 D_2 + C D_1 D_2\right) \quad \text{(S3.2)}$$

With the assumption that $D_1, D_2 > 0$ (which is expected since these are supposed to the sums of neural activity), we take the partial derivatives of Eq (S3.2) to obtain the polynomials in Eq (15).
