# Supplementary material for: Modeling the diverse effects of divisive normalization on noise correlations
Source: PLoS Comput Biol. 2023 Nov 30;19(11):e1011667. doi: 10.1371/journal.pcbi.1011667 (PMC10715670; doi:10.1371/journal.pcbi.1011667)
Supplement: S4 Text — Examining model performance on deconvolved fluorescence traces. (PDF) [file pcbi.1011667.s004.pdf]

S4 Text

Analysis of Deconvolved Imaging Data

In the main paper, we chose to analyze the raw normalized fluorescence traces (see Methods for details of data analysis and Discussion for our rationale). However, as deconvolution of calcium traces are widely used in the literature, we also performed our analysis on the data processed with OASIS deconvolution [1]. We analyzed the data using two different constraints during the spike inference: 1) only constraining the resulting deconvolved traces to be positive (*suite2p*) [2], and 2) constraining the deconvolved traces to be sparse (*ℓ_1 OASIS*) [3]. Using deconvolution also increased the number of neurons that were assessed to be visually responsive: from the raw fluorescence traces, we assessed that 295 neurons were visually responsive (see Methods subsection Data Collection and Processing), while for suite2p there were 688 neurons and for ℓ_1 OASIS there were 856 neurons. For comparison of the deconvolutional methods to the normalized fluorescence traces, we only analyze neurons and neuronal pairs that were considered in the main text.

First, we examined how deconvolution would change the statistics of the neuronal data, in particular how deconvolution would alter the distribution of noise correlations. From Fig Aa, we see that, on average, the changes of noise correlations with contrast are similar across each of the three preprocessing methods. The main difference among the three methods is the magnitude of the noise correlations. On average, noise correlations appear to increase with contrast for all three preprocessing methods except for a decrease in correlation strength for 80% contrast level. One possible reason for this decrease is that, for two of the recording sessions animals were not presented with a grating with this contrast level, leading to less data relative to the other contrast levels. The distributions of noise correlations are not identical, however, as seen in Fig Ab. While the normalized traces and suite2p deconvolution appear very similar, the ℓ_1 OASIS deconvolved traces have more peaked noise correlations near 0 and skewed towards negative noise correlations. This difference in distribution may be due

to the sparsity constraint inherent to this method, which would decrease the overall neuronal activity relative to the normalized traces and suite2p.

Next, we looked at how well the RoG and the Modulated Gaussian (MG) model fit to the deconvolved data (Fig Ac; see Methods subsection Model Comparison and Results subsection Pairwise Ratio of Gaussians Model Captures Correlated Variability in Mouse V1 for the corresponding analysis using the normalized calcium traces). We see that, for suite2p, there is only a marginal improvement in goodness of fit for the RoG relative to the MG, while for ℓ_1 OASIS the RoG outperformed the MG, as was found in the normalized calcium traces (Fig 5C-D in the main text).

References

[1] Friedrich J, Zhou P, Paninski L. Fast Online Deconvolution of Calcium Imaging Data. PLOS Computational Biology. 2017;13(3):1–26. doi:10.1371/journal.pcbi.1005423.

[2] Pachitariu M, Stringer C, Dipoppa M, Schröder S, Rossi LF, Dalgleish H, et al. Suite2p: Beyond 10,000 Neurons with Standard Two-Photon Microscopy. bioRxiv : the preprint server for biology. 2017;doi:10.1101/061507.

[3] Lyall EH, Mossing DP, Pluta SR, Chu YW, Dudai A, Adesnik H. Synthesis of a Comprehensive Population Code for Contextual Features in the Awake Sensory Cortex. eLife. 2021;10:e62687. doi:10.7554/eLife.62687.

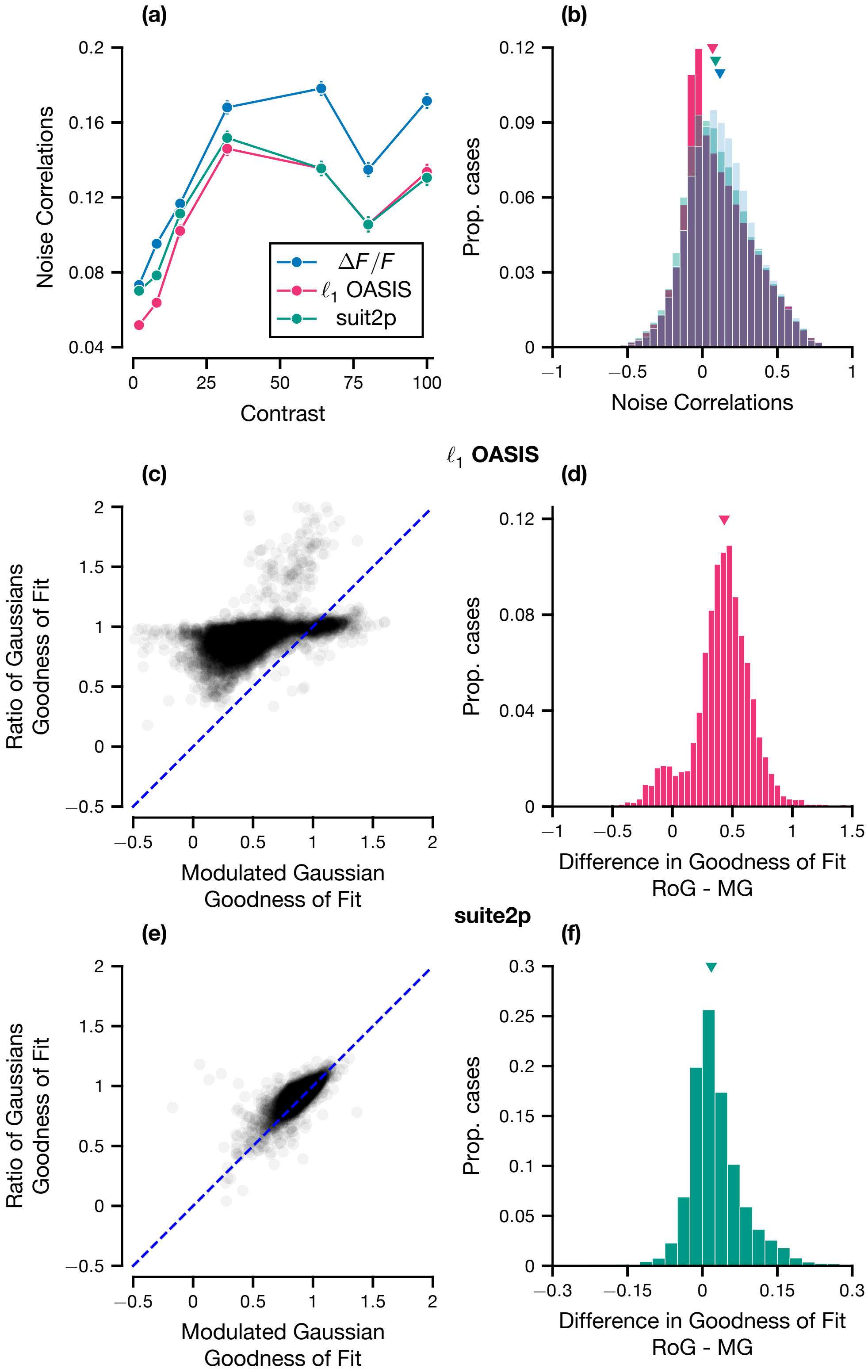

Figure A *(caption on next page)*

Figure A: Comparison of Raw and Deconvolved Data
(a) Tuning of noise correlations with contrasts across all recorded pairs. The error bars are the standard error of the mean. (b) Histogram of noise correlations across all contrast levels. (c-f) Goodness of fit comparison for the RoG and the MG models, like Fig 5C-D for suite2p (c,d) and ℓ_1 OASIS (e,f)
