# Supplementary material for: Modeling the diverse effects of divisive normalization on noise correlations
Source: PLoS Comput Biol. 2023 Nov 30;19(11):e1011667. doi: 10.1371/journal.pcbi.1011667 (PMC10715670; doi:10.1371/journal.pcbi.1011667)
Supplement: S5 Text — Mathematical derivations relating noise correlations and normalization in the model. (PDF) [file pcbi.1011667.s005.pdf]

S5 Text

S1 Derivation of Relationship Between Mean Normalization Strength and Noise Correlations

(see the Results subsection Modulations of Correlated Variability Depend on Sharing of Normalization in the main text)

We use the same notation as in the main text (see subsection Generative Model – Pairwise Ratio of Gaussians (RoG) and Eq (7) in the main text). The true characterization of how changes in the mean normalization strengths (μ_{D_1}, μ_{D_2}) cause changes in $\rho_{NC} := \mathrm{Corr}(R_1, R_2)$ (Eq (7)) would be to consider the directional derivative $\nabla \rho_{NC} \cdot (1, 1)$. However, it will suffice to consider the partial derivative with respect to either μ_{D_1} or μ_{D_2}. We note a few identities which are easy to verify:

$$\frac{\partial \delta_D}{\partial \mu_D} = \left(\frac{\beta_D}{2} - 1\right)\frac{\delta_D}{\mu_D} \qquad \widehat{\sigma_R}^2 := \delta_N^2 + \delta_D^2 + \widehat{\sigma_\eta}^2$$

$$\frac{\partial \widehat{\sigma_\eta}}{\partial \mu_D} = \frac{\widehat{\sigma_\eta}}{\mu_D} \qquad \frac{\partial \widehat{\sigma_R}^2}{\partial \mu_D} = (\beta_D - 2)\frac{\delta_D^2}{\mu_D} + 2\frac{\widehat{\sigma_\eta}^2}{\mu_D}$$

Then considering $\frac{\partial \rho_{NC}}{\partial \mu_{D_1}}$, we compute using the chain rule:

$$\begin{aligned}
\frac{\partial \rho_{NC}}{\partial \mu_{D_1}} &= (\rho_N \delta_{N_1}\delta_{N_2} + \rho_D \delta_{D_1}\delta_{D_2} + \rho_\eta \widehat{\sigma_{\eta_1}}\widehat{\sigma_{\eta_2}})\frac{\partial}{\partial \mu_{D_1}}(\widehat{\sigma_{R_1}}^2\widehat{\sigma_{R_2}}^2)^{-1/2} \\
&\quad + \frac{\rho_D \frac{\partial \delta_{D_1}}{\partial \mu_{D_1}}\delta_{D_2} + \rho_\eta \frac{\partial \widehat{\sigma_{\eta_1}}}{\partial \mu_{D_1}}\widehat{\sigma_{\eta_2}}}{(\widehat{\sigma_{R_1}}^2\widehat{\sigma_{R_2}}^2)^{1/2}} \\
&= (\rho_N \delta_{N_1}\delta_{N_2} + \rho_D \delta_{D_1}\delta_{D_2} + \rho_\eta \widehat{\sigma_{\eta_1}}\widehat{\sigma_{\eta_2}})(-\frac{1}{2}(\widehat{\sigma_{R_1}}^2\widehat{\sigma_{R_2}}^2)^{-3/2})\widehat{\sigma_{R_2}}^2 \left((\beta_{D_1} - 2)\frac{\delta_{D_1}^2}{\mu_{D_1}} + 2\frac{\widehat{\sigma_{\eta_1}}^2}{\mu_{D_1}}\right) \\
&\quad + \frac{\rho_D \left(\frac{\beta_{D_1}}{2} - 1\right)\frac{\delta_{D_1}}{\mu_{D_1}}\delta_{D_2} + \rho_\eta \frac{\widehat{\sigma_{\eta_1}}}{\mu_{D_1}}\widehat{\sigma_{\eta_2}}}{(\widehat{\sigma_{R_1}}^2\widehat{\sigma_{R_2}}^2)^{1/2}}
\end{aligned}$$

Simplifying, we get the equation:

$$\frac{\partial \rho_{NC}}{\partial \mu_{D_1}} = \frac{1}{\mu_{D_1}\widehat{\sigma_{R_1}}\widehat{\sigma_{R_2}}}\Bigg[(\rho_N\delta_{N_1}\delta_{N_2} + \rho_D\delta_{D_1}\delta_{D_2} + \rho_\eta\widehat{\sigma_{\eta_1}}\widehat{\sigma_{\eta_2}})\left(\frac{\left(1-\frac{\beta_{D_1}}{2}\right)\delta_{D_1}^2 - \widehat{\sigma_{\eta_1}}^2}{\widehat{\sigma_{R_1}}^2}\right) \tag{S5.1}$$

$$+ \left(\frac{\beta_D}{2} - 1\right)\rho_D\delta_{D_1}\delta_{D_2} + \rho_\eta\widehat{\sigma_{\eta_1}}\widehat{\sigma_{\eta_2}}\Bigg]$$

To reproduce the patterns noted in Fig 2A, we first set $\eta \equiv 0$ and $\rho_N = 0, \rho_D > 0$. Then

$$\begin{aligned}\frac{\partial \rho_{NC}}{\partial \mu_{D_1}} &= \frac{1}{\mu_{D_1}\widehat{\sigma_{R_1}}\widehat{\sigma_{R_2}}}\left((\rho_D\delta_{D_1}\delta_{D_2})\left(\frac{\left(1-\frac{\beta_{D_1}}{2}\right)\delta_{D_1}^2}{\widehat{\sigma_{R_1}}^2} + \left(\frac{\beta_{D_1}}{2} - 1\right)\rho_D\delta_{D_1}\delta_{D_2}\right)\right.\\ &= \frac{\rho_D\delta_{D_1}\delta_{D_2}}{\mu_{D_1}\widehat{\sigma_{R_1}}\widehat{\sigma_{R_2}}}\left(1-\frac{\beta_{D_1}}{2}\right)\left(\frac{\delta_{D_1}^2}{\widehat{\sigma_{R_1}}^2} - 1\right) < 0\end{aligned} \tag{S5.2}$$

Because $\beta_{D_1} < 2 \Rightarrow 1 - \frac{\beta_{D_1}}{2} > 0$. Moreover, $\widehat{\sigma_{R_1}}^2 - \delta_{D_1}^2 = \delta_{N_1}^2 > 0 \Rightarrow \frac{\delta_{D_1}^2}{\widehat{\sigma_{R_1}}^2} < 1$. Thus, ρ_{NC} decreases as a function of normalization strength.

Similarly, when $\eta \equiv 0, \rho_D = 0, \rho_N > 0$ (Fig 2B), then

$$\frac{\partial \rho_{NC}}{\partial \mu_{D_1}} = \frac{\rho_N\delta_{N_1}\delta_{N_2}}{\mu_{D_1}\widehat{\sigma_{R_1}}\widehat{\sigma_{R_2}}}\left(1-\frac{\beta_{D_1}}{2}\right)\frac{\delta_{D_1}^2}{\widehat{\sigma_{R_1}}^2} > 0 \tag{S5.3}$$

Demonstrating that ρ_{NC} increases with normalization in this case.

When ρ_N, ρ_D are the opposite signs, the inequalities in (Eq S5.2) and (Eq S5.3) reverse and the relationship between ρ_{NC} and normalization switch to be increasing and decreasing, respectively.

Next, when $\eta \equiv 0$ and $\rho_N = -\rho_D = \rho$, (S5.1) reduces to:

$$\frac{\partial \rho_{NC}}{\partial \mu_{D_1}} = \rho\frac{1}{\mu_{D_1}\widehat{\sigma_{R_1}}\widehat{\sigma_{R_2}}}\left(1-\frac{\beta_{D_1}}{2}\right)\left(\delta_{D_1}\delta_{D_2}\left(1-\frac{\delta_{D_1}^2}{\widehat{\sigma_{R_1}}^2}\right) + \delta_{N_1}\delta_{N_2}\frac{\delta_{D_1}^2}{\widehat{\sigma_{R_1}}^2}\right) \tag{S5.4}$$

Thus, when $\rho > 0 \Rightarrow \frac{\partial \rho_{NC}}{\partial \mu_{D_1}} > 0$. In fact, we can use Eq (S5.4) along with Eqs (S5.2) and (S5.3) to generalize to all cases where ρ_N, ρ_D have opposite signs. As an example, suppose $\rho_D < 0$ and $\rho_N = |\rho_D| + C > 0, C > 0$. Then we can decompose $\frac{\partial \rho_{NC}}{\partial \mu_{D_1}}$ as a combination of Eq (S5.4), with $\rho = |\rho_D|$ and Eq (S5.3), with $\rho_N = C > 0$. Since both these terms are positive, we conclude that ρ_{NC} increases with normalization when $\rho_N > 0, \rho_D < 0, \rho_N > |\rho_D|$.

Lastly, we consider when $\eta \not\equiv 0$. The nature of Eq (S5.1) prevents us from making any conclusions in general, as the sign of $\frac{\partial \rho_{NC}}{\partial \mu_{D_1}}$ will depend on the magnitudes of the various terms, similar to when $\rho_N, \rho_D > 0$. To get some understanding of the behavior of ρ_{NC}, let us assume that $\rho_\eta = 0, \rho_D = 0$, a generalization of Eq (S5.3). For these assumptions, we have

$$\frac{\partial \rho_{NC}}{\partial \mu_{D_1}} = \frac{\rho_N \delta_{N_1} \delta_{N_2}}{\mu_{D_1} \widehat{\sigma_{R_1}} \widehat{\sigma_{R_2}}} \left(\left(1 - \frac{\beta_{D_1}}{2}\right) \frac{\delta_{D_1}^2}{\widehat{\sigma_{R_1}}^2} - \frac{\widehat{\sigma_{\eta_1}}^2}{\widehat{\sigma_{R_1}}^2} \right) \tag{S5.5}$$

Here, if $\frac{\widehat{\sigma_{\eta_1}}^2}{\widehat{\sigma_{R_1}}^2} \approx 0$ (i.e., $\widehat{\sigma_{\eta_1}}^2 << \widehat{\sigma_{R_1}}^2$), then Eq (S5.5) should be approximately equal to Eq (S5.3). This gives the heuristic that if the residual variance is much less than the variance of the neural response, then the presence of this noise should have little effect on these derived relationships.
