# Supplementary material for: Modeling the diverse effects of divisive normalization on noise correlations
Source: PLoS Comput Biol. 2023 Nov 30;19(11):e1011667. doi: 10.1371/journal.pcbi.1011667 (PMC10715670; doi:10.1371/journal.pcbi.1011667)
Supplement: S6 Text — Additional considerations for ρ parameter estimation from data. (PDF) [file pcbi.1011667.s006.pdf]

S6 Text

Further Discussion of Parameter Identifiability

In the Results subsection Inference of Correlation Parameters and the Discussion, we investigated and discussed the accuracy of the estimation of model parameters, namely the ρ parameters. We hypothesize that the inability for the optimization to capture the magnitude of these parameters is due to the parametrization of the variance of the numerators and denominators and how they interact with the ρ parameters in the equation for the noise correlations (Eq (7)). In the ensuing discussion, we focus on one possible multiplicative interaction between model parameters that may impede exact inference of the ρ parameters to give intuition about the problem. In full generality, there are many multiplicative interactions involving the ρ parameters in Eq (7), including the mean of the numerator and/or denominator variables raised to a power (depending on the parameter β) and the variances of the neural responses, which are functions of the means and variances for the numerators and denominators (Eq (6)). Additionally, as the means of the the numerators and denominators involve further parameters describing the base normalization model (e.g., the contrast response function, Eq (8)), it would be difficult to fully describe all the possible multiplicative interactions involving the ρ parameters and other model parameters that are fit to data.

As a simplified example of this phenomenon, consider the expression for correlation in the RoG (Eq (7)) with $\rho_D = 0, \rho_\eta = 0$, all $\boldsymbol{\beta} = 2$, and $\sigma_{R_1} = \sigma_{R_2} = 1$ (this can be achieved manually tuning $\sigma_{\eta_1}, \sigma_{\eta_2}$), then we have that $\text{Corr}(R_1, R_2) \propto \rho_N \sqrt{\alpha_{N_1}} \sqrt{\alpha_{N_2}}$. This effectively partitions a single number (the noise correlation) into three different contributions, which makes it difficult to correctly infer the magnitude of each parameter. In the two-step optimization procedure we use in this paper (see Methods subsection Fitting the RoG to Data), these parameters are estimated during the first phase in which correlations are ignored. If these parameters are incorrectly estimated to be at the extremes of the broad parameter

bounds, when the ρ parameters are estimated in the second phase of the optimization, they will be forced to the extremes of their ranges to compensate. Using our previous example, suppose that $\boldsymbol{\alpha}$ are all (incorrectly) estimated to be 0.1. If the noise correlation is measured to be 0.1, this will force $\rho_N = 1$. This is an extreme example, but is illustrative of the issue with estimating these parameters. This problem becomes compounded by the presence of the other ρ parameters needing to be optimized.

One solution to this problem would be to put tighter constraints on the model parameters, primarily the $\boldsymbol{\alpha}, \boldsymbol{\beta}$ parameters as the parameters of the base normalization model (e.g., the contrast response function, Eq (8)) are more explicitly constrained by the data. The bound constraints for $\boldsymbol{\alpha}, \boldsymbol{\beta}$ are hyperparameters that may need to be tuned explicitly. It is *a priori* difficult to constrain the $\boldsymbol{\alpha}, \boldsymbol{\beta}$ parameters as they represent the mean-variance dependence for internal variables of the model (numerator and denominator) which are not measurable. We currently have a very broad range of $\boldsymbol{\alpha} \in [0.1, 20]$; the range of $\boldsymbol{\beta} \in [1, 2]$ is more restricted under the assumption that the numerator and denominator variables should overdispersed, as is often found in neuronal data ([1] but see [2, 3]). One possible additional constraint would be to replace the eight $\boldsymbol{\alpha}, \boldsymbol{\beta}$ parameters with two global parameters, $\boldsymbol{\alpha} = a, \boldsymbol{\beta} = b$ for some a, b, or have separate parameters for the numerators and denominators ($\boldsymbol{\alpha_N} = a_N, \boldsymbol{\beta_N} = b_N$ and similarly for the denominator). These parameters could also be optimized, or they could be fixed to a specific value; for instance, by setting $\boldsymbol{\alpha} = 1$, which would match the mean-variance relationship found in some neural recordings. A related approach would be to constrain ρ_N using measurements of mean firing rate tuning similarity (see end of Methods subsection Generative Model - Pairwise Ratio of Gaussians (RoG)). Complementary to further constraining the model by directly changing the parameter bounds or dependence among variables discussed above, we can explore global optimization algorithms to improve parameter estimates or modified objective functions, namely by imposing some sort of regularization on the parameters.

References

[1] Goris RLT, Movshon JA, Simoncelli EP. Partitioning Neuronal Variability. Nature Neuroscience. 2014;17(6):858–865. doi:10.1038/nn.3711.

[2] Gao Y, Busing L, Shenoy KV, Cunningham JP. High-Dimensional Neural Spike Train Analysis with Generalized Count Linear Dynamical Systems. In: Cortes C, Lawrence N, Lee D, Sugiyama M, Garnett R, editors. Advances in Neural Information Processing Systems. vol. 28. Curran Associates, Inc.; 2015.

[3] Stevenson IH. Flexible Models for Spike Count Data with Both Over- and under- Dispersion. Journal of Computational Neuroscience. 2016;41(1):29–43. doi:10.1007/s10827-016-0603-y.
