# Supplementary material for: Modeling the diverse effects of divisive normalization on noise correlations
Source: PLoS Comput Biol. 2023 Nov 30;19(11):e1011667. doi: 10.1371/journal.pcbi.1011667 (PMC10715670; doi:10.1371/journal.pcbi.1011667)
Supplement: S7 Text — Comparison of pairwise and independent Ratio of Gaussians model goodness of fits. (PDF) [file pcbi.1011667.s007.pdf]

# S7 Text

## Pairwise Model Outperforms the Independent Model in Simulations and V1 data When Noise Correlations are Large

Our previous work [1] demonstrated that the independent RoG model provided an excellent fit to single-neuron data. Moreover, by construction, the independent and pairwise model best-fit parameters are identical except for the correlation parameters (the $\rho$ parameters, see Methods subsection Fitting the RoG to Data). Thus, we expected any differences in the fit quality (as measured by the negative log-likelihood using the best fit parameters) between the independent and pairwise RoG models to be minimal for pairs of neurons with noise correlations close to zero, and that the pairwise RoG would outperform the independent RoG for pairs of neurons with prominent correlated variability.

To test these predictions, we simulated pairs of neurons with parameters selected as described in Methods (see Methods subsection Generating Realistic Pairwise Neural Activity from the Model) and compared the negative log-likelihood values for the pairwise and independent model (a lower negative log-likelihood denotes better goodness of fit). For these simulations, we used 6 stimulus contrasts ($[6.25, 12.5, 25, 50, 100]$) and 1000 trials. We used cross-validation to account for the extra free parameters of the pairwise model (details in Methods subsection Fitting the RoG to Data). We found that 5443/11628 of the pairs were better captured by the pairwise model, and on average the pairwise model slightly outperformed the independent model (mean percent difference = 0.964, $p<0.01$). We studied how the difference of likelihoods depends on the noise correlation of each pair (median noise correlation across contrasts; Fig Aa). We found that, for most pairs of neurons with magnitude of noise correlations greater than 0.1, the pairwise model outperformed the independent model (3411/4040 pairs). To quantify the dependence of this model improvement on noise correlation strength, we binned simulated pairs of neurons by the magnitude of noise cor-

relations and computed the average difference of negative log-likelihoods for the pairwise and independent models (Fig Ab). This demonstrates that, as the magnitude of the noise correlations increases, the pairwise model provides an increasingly better fit to the data over the independent model.

We further validated this synthetic result using the calcium fluorescence data and obtained similar results (Fig Ac-d; see Methods subsection Data Collection and Processing).

## References

[1] Coen-Cagli R, Solomon SS. Relating Divisive Normalization to Neuronal Response Variability. The Journal of Neuroscience. 2019;39(37):7344–7356. doi:10.1523/JNEUROSCI.0126-19.2019.

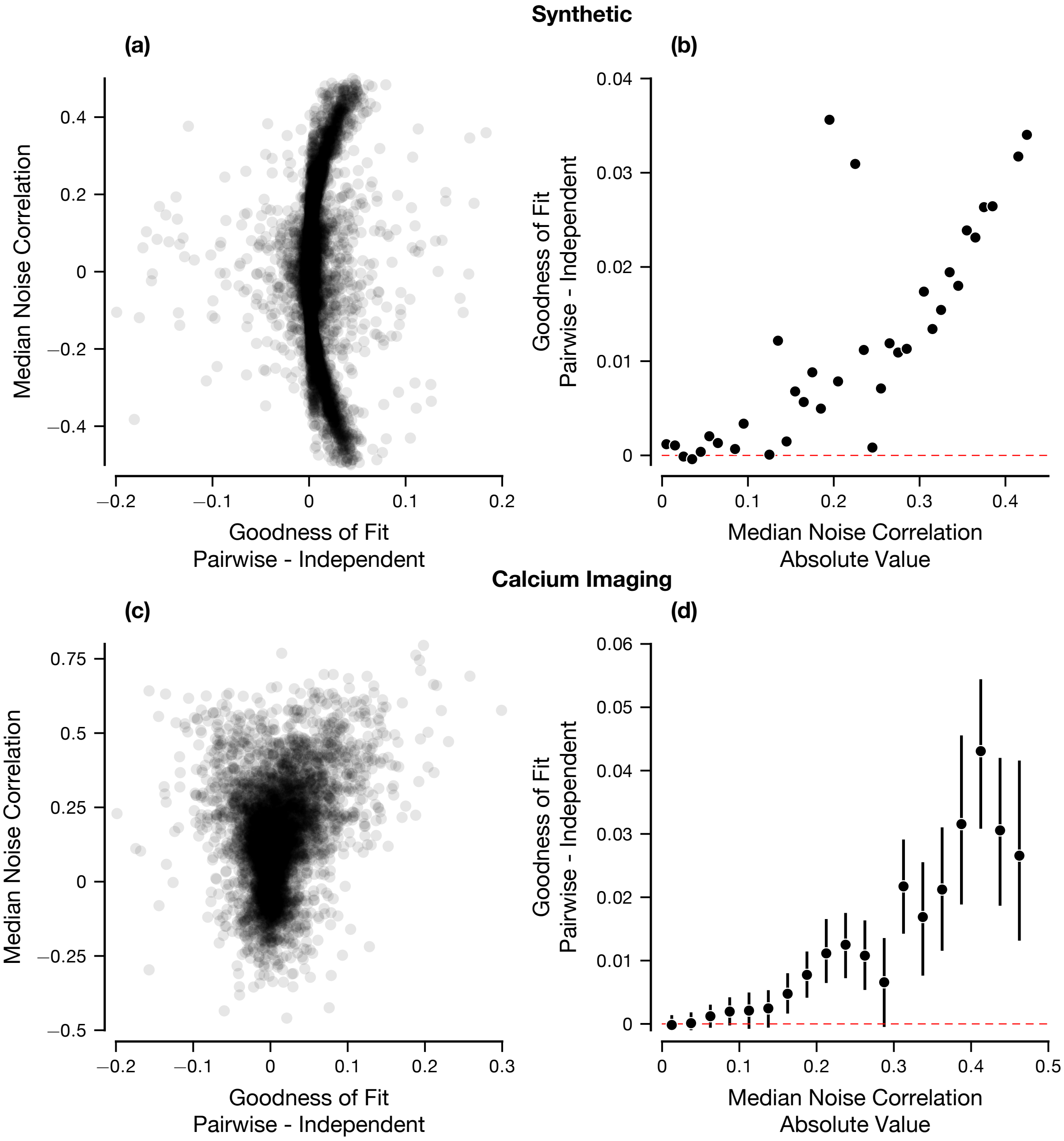


**Figure A: Improvement in Fit of the Pairwise RoG over the Independent RoG Depends on the Magnitude of the Noise Correlations**

(a) Scatter plot demonstrating the dependence of the difference in fit quality on the median noise correlation (across contrasts). (b) Mean percent difference when binning by the magnitude of the median noise correlations. Data were generated as described in Methods subsection Generating Realistic Pairwise Neural Activity from the Model. (c) Scatter plot of the difference in goodness of fit score of the Pairwise and Independent RoG when applied to calcium imaging data (see Results subsection Pairwise Ratio of Gaussians Model Captures Correlated Variability in Mouse V1). (d) Mean difference in goodness of fit between Pairwise and Independent RoG binning by the magnitude of the median noise correlations in calcium imaging data. Error bars are bootstrapped 95% confidence intervals.
