# Supplementary material for: Modeling the diverse effects of divisive normalization on noise correlations
Source: PLoS Comput Biol. 2023 Nov 30;19(11):e1011667. doi: 10.1371/journal.pcbi.1011667 (PMC10715670; doi:10.1371/journal.pcbi.1011667)
Supplement: S1 Fig — Noise correlations in the model (Eq (7) in Methods subsection Generative model—pairwise Ratio of Gaussians (RoG)) can be modulated by stimulus strength (i.e., contrast), the correlation parameters of the model (ρN, ρD) and the parameters of the normalization model, in this case (R1max,R2max) and (ϵ1, ϵ2) (see Fig 2). To understand these effects in isolation, we looked at how noise correlations (Eq (7)) changed with respect to each parameter, while keeping the other parameters constant. We illustrate with noise correlations that increase with contrast (A1-E1), and correlations that decrease with contrast (A2-E2). (A) Dependence of noise correlations on contrast. Three contrast levels that are fixed in the other panels are shown. (B) Dependence of noise correlations on ρN. (C) Dependence of noise correlations on ρD. (D) Dependence of noise correlations on (R1max,R2max), shown as a contour plot with the shade of color indicating noise correlation level. Different colors indicate different contrast levels as shown in the legend. (E) Dependence of noise correlations on (ϵ1, ϵ2). (A1-E1) uses the following parameters (when not fixed): (R1max,R2max)=(25,50),(ϵ1,ϵ2)=(10,25),(ρN,ρD)=(0,0.5)(αN1,αN2)=(αD1,αD2)=(1,1),(βN1,βN2)=(βD1,βD2)=(1.5,1.5) (A2-E2) uses the same parameters except with (ρN, ρD) = (0.5, 0). Contrast levels were {1,…,100}. (PDF) [file pcbi.1011667.s008.pdf]

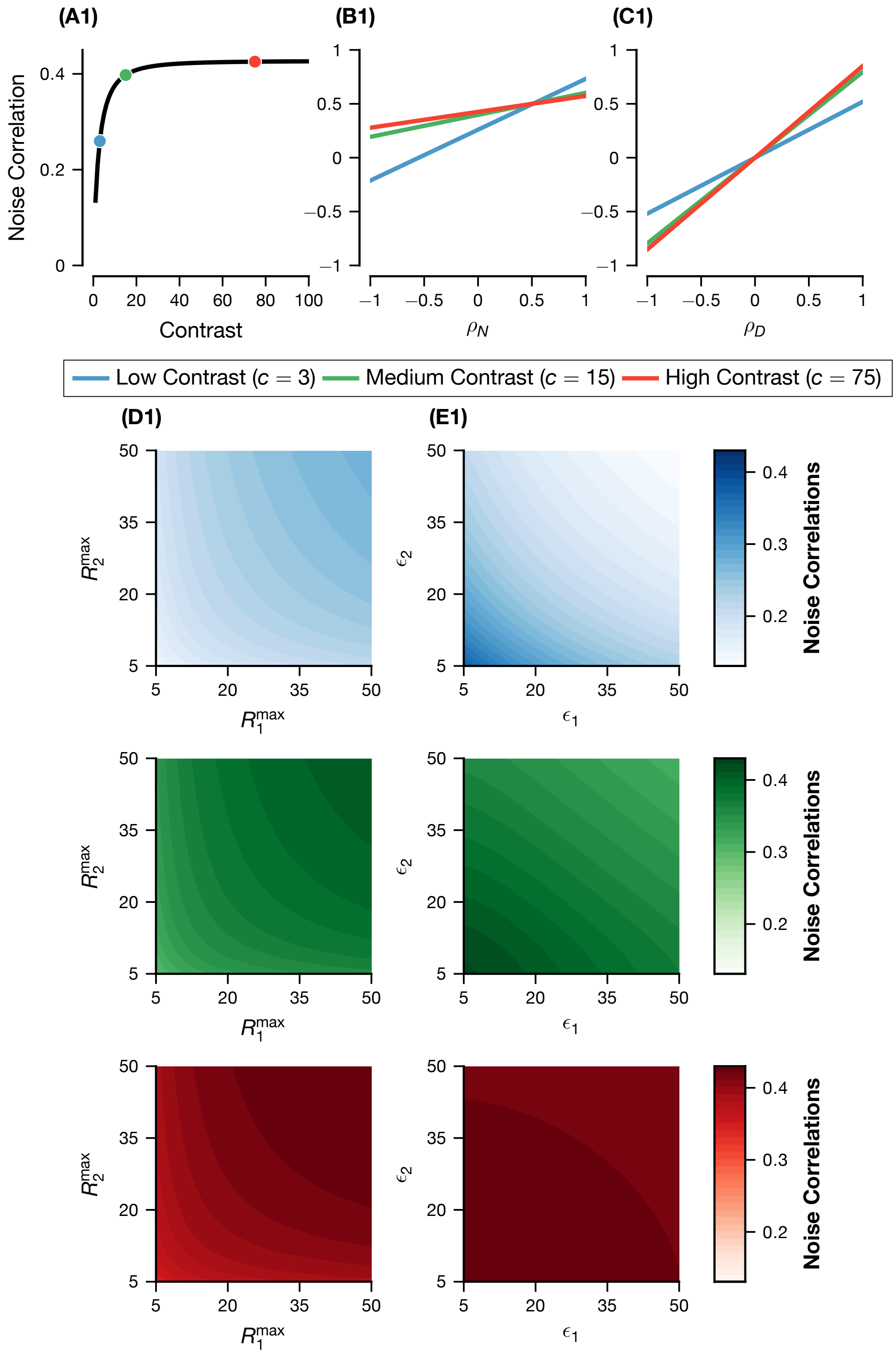

Figure S1 *(caption on next page)*

Figure S1: Relationship Between Noise Correlations and Ratio of Gaussians Parameters
Noise correlations in the model (Eq (7) in Methods subsection Generative Model – Pairwise Ratio of Gaussians (RoG)) can be modulated by stimulus strength (i.e., contrast), the correlation parameters of the model (ρ_N, ρ_D) and the parameters of the normalization model, in this case $(R_1^{\max}, R_2^{\max})$ and (ϵ_1, ϵ_2) (see Fig 2). To understand these effects in isolation, we looked at how noise correlations (Eq (7)) changed with respect to each parameter, while keeping the other parameters constant. We illustrate with noise correlations that increase with contrast (A1-E1), and correlations that decrease with contrast (A2-E2). (A) Dependence of noise correlations on contrast. Three contrast levels that are fixed in the other panels are shown. (B) Dependence of noise correlations on ρ_N. (C) Dependence of noise correlations on ρ_D. (D) Dependence of noise correlations on $(R_1^{\max}, R_2^{\max})$, shown as a contour plot with the shade of color indicating noise correlation level. Different colors indicate different contrast levels as shown in the legend. (E) Dependence of noise correlations on (ϵ_1, ϵ_2).
(A1-E1) uses the following parameters (when not fixed): $(R_1^{\max}, R_2^{\max}) = (25, 50), (\epsilon_1, \epsilon_2) = (10, 25), (\rho_N, \rho_D) = (0, 0.5)(\alpha_{N_1}, \alpha_{N_2}) = (\alpha_{D_1}, \alpha_{D_2}) = (1, 1), (\beta_{N_1}, \beta_{N_2}) = (\beta_{D_1}, \beta_{D_2}) = (1.5, 1.5)$ (A2-E2) uses the same parameters except with $(\rho_N, \rho_D) = (0.5, 0)$. Contrast levels were {1,...,100}.

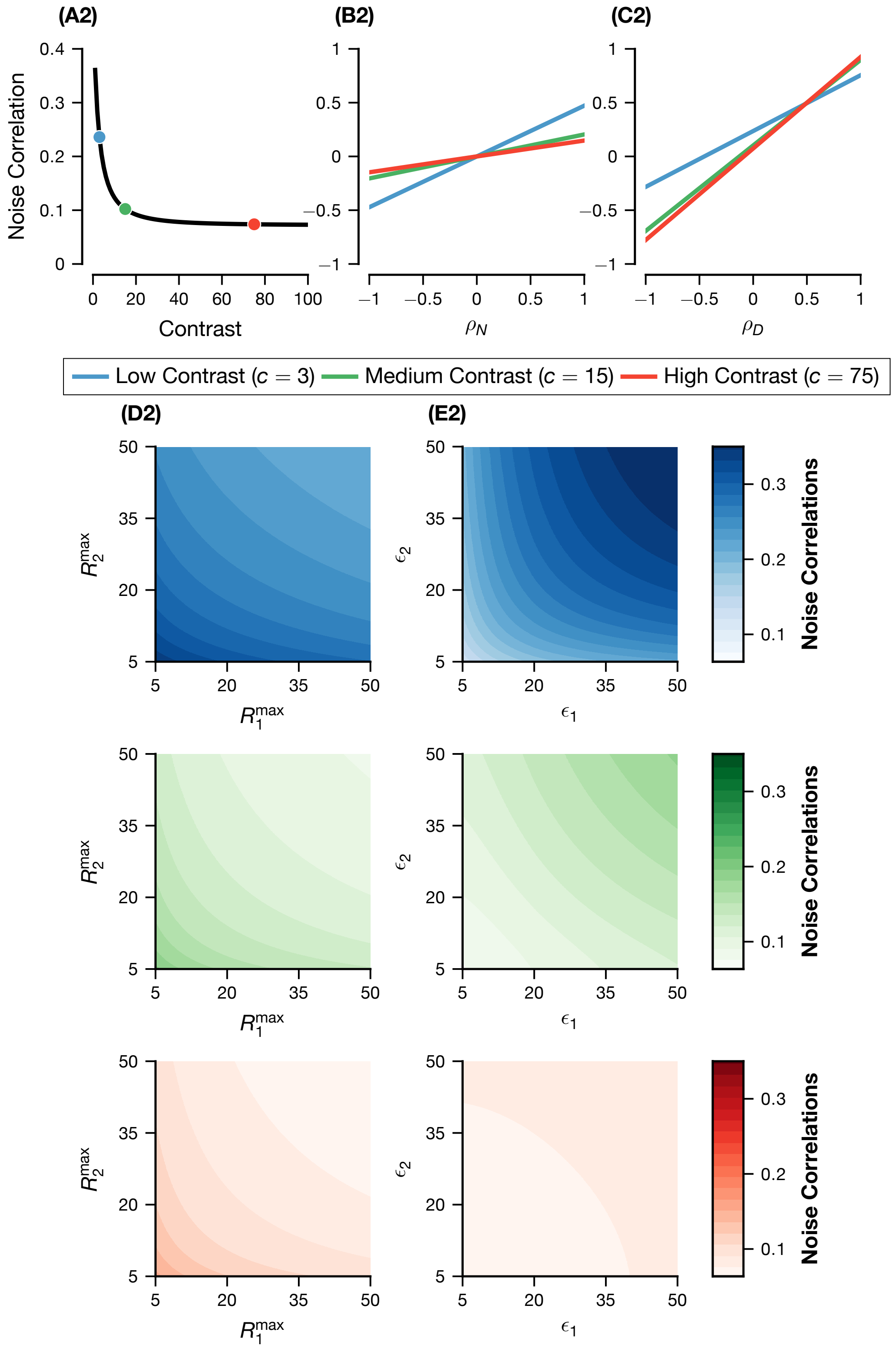

Continuation of **Figure S1**
