# Supplementary material for: Modeling the diverse effects of divisive normalization on noise correlations
Source: PLoS Comput Biol. 2023 Nov 30;19(11):e1011667. doi: 10.1371/journal.pcbi.1011667 (PMC10715670; doi:10.1371/journal.pcbi.1011667)
Supplement: S2 Fig — Expands upon Fig 2 (see Results subsection Modulations of correlated variability depend on sharing of normalization) to include cases where (ρN, ρD) can be negative or have opposite signs. Figure was created using the exact same method and synthetic dataset as Fig 2: see the caption in the main text for details. (PDF) [file pcbi.1011667.s009.pdf]

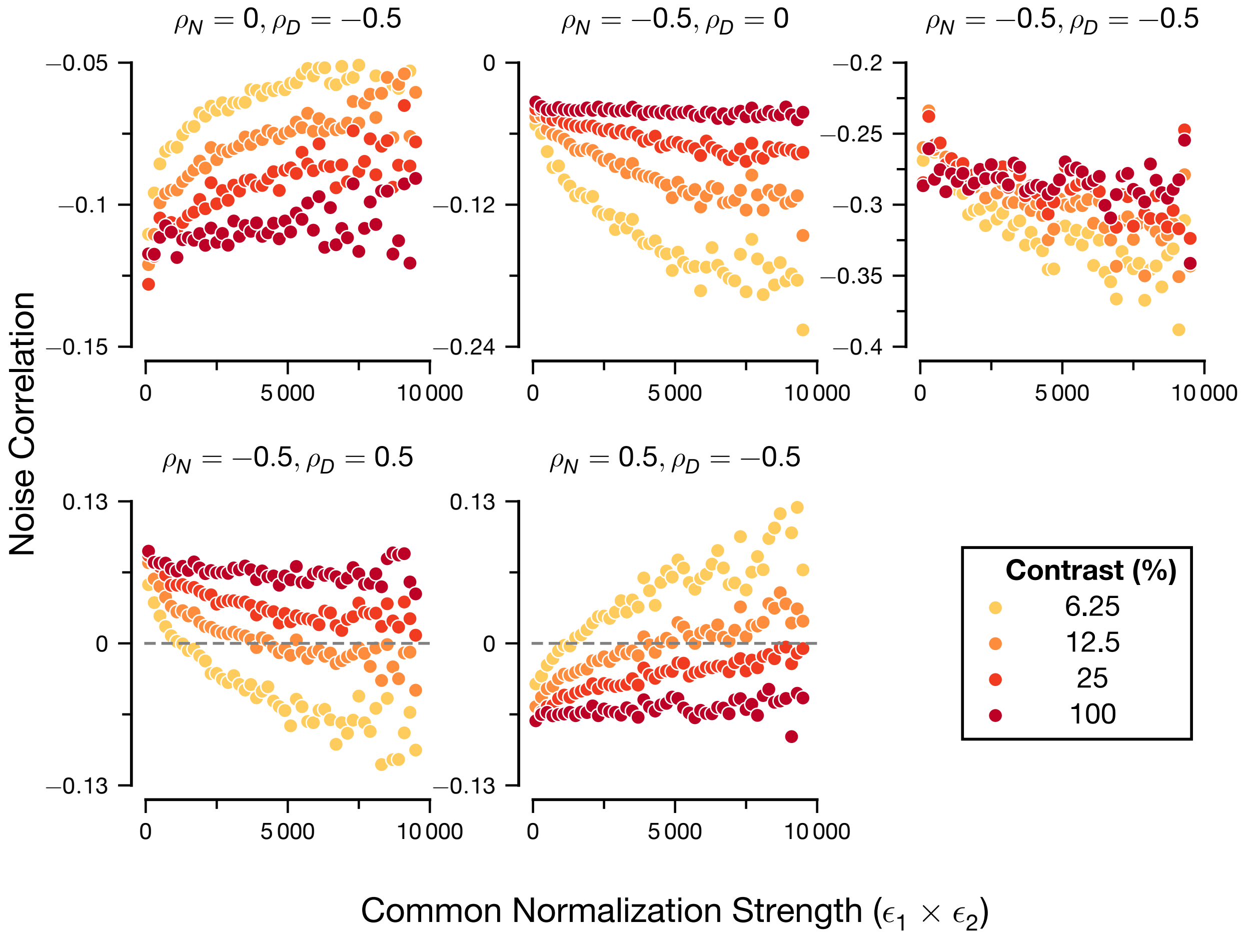

Figure S2: Relationship Between Noise Correlations and Denominator Strength
Expands upon Fig 2 (see Results subsection Modulations of Correlated Variability Depend on Sharing of Normalization) to include cases where (ρ_N, ρ_D) can be negative or have opposite signs. Figure was created using the exact same method and synthetic dataset as Fig 2: see the caption in the main text for details.
